# Dynamic Changes in Neuroglial Reaction and Tissue Repair after Photothrombotic Stroke in Neonatal Mouse

**DOI:** 10.3390/brainsci14020152

**Published:** 2024-02-01

**Authors:** Yitong Liu, Pifang Gong, Guibo Qi, Han Tang, Runshan Gui, Congcong Qi, Song Qin

**Affiliations:** 1 Department of Anatomy, Histology and Embryology, School of Basic Medical Sciences, Fudan University, Shanghai 200032, China; 2 Department of Laboratory Animal Science, Fudan University, Shanghai 200032, China; 3 State Key Laboratory of Medical Neurobiology and MOE Frontiers Center for Brain Science, Fudan University, Shanghai 200032, China

**Keywords:** photothrombotic stroke, ischemic stroke, neonatal mouse, glial border

## Abstract

Perinatal and neonatal ischemic stroke is a significant cause of cognitive and behavioral impairments. Further research is needed to support models of neonatal ischemic stroke and advance our understanding of the mechanisms of infarction formation following such strokes. We used two different levels of photothrombotic stroke (PTS) models to assess stroke outcomes in neonatal mice. We measured brain damage, dynamic changes in glial cells, and neuronal expression at various time points within two weeks following ischemic injury. Our results from 2,3,5-Triphenyltetrazolium chloride (TTC) staining and immunofluorescence staining showed that in the severe group, a dense border of astrocytes and microglia was observed within 3 days post infarct. This ultimately resulted in the formation of a permanent cortical cavity, accompanied by neuronal loss in the surrounding tissues. In the mild group, a relatively sparse arrangement of glial borders was observed 7 days post infarct. This was accompanied by intact cortical tissue and the restoration of viability in the brain tissue beyond the glial boundary. Additionally, neonatal ischemic injury leads to the altered expression of key molecules such as Aldh1L1 and Olig2 in immature astrocytes. In conclusion, we demonstrated the dynamic changes in glial cells and neuronal expression following different degrees of ischemic injury in a mouse model of PTS. These findings provide new insights for studying the cellular and molecular mechanisms underlying neuroprotection and neural regeneration after neonatal ischemic injury.

## 1. Introduction

Perinatal stroke, also known as neonatal stroke, refers to various types of focal neurological injuries that occur during early brain development and can lead to significant death and disability [1]. The incidence of perinatal stroke is estimated to range from 1 in 1600 to 1 in 3000 births, potentially affecting over 5 million people worldwide [2,3]. Neonatal ischemia, a common cause of brain stroke in newborns, can result in cerebral palsy, learning disabilities, and epilepsy [4,5]. However, there is limited evidence regarding the pathophysiology and cytology of neonatal ischemia, and effective methods for preventing and treating perinatal ischemic stroke patients are currently lacking.

Emerging evidence indicates significant differences in the mechanisms of ischemic injury between immature and adult brains [6,7]. Further research on neonatal ischemic stroke mechanisms requires robust support from animal models. While various models have been adapted to simulate injuries in newborns, none fully replicate the microenvironment of human neonatal stroke [8]. The photothrombotic stroke (PTS) model, a minimally invasive and highly reproducible model of cerebral small vessel occlusion, allows for detailed investigation into the cellular and molecular mechanisms underlying ischemic injury [9,10,11]. However, there is a lack of available description regarding the pathological effects of the PTS model in neonatal mice.

Under various pathological conditions of ischemic injury, neuroglial cells display varied pathophysiological functions at specific temporal windows, including damage repair and the maintenance of local microenvironment homeostasis, and certain aspects of neuroglial reactions might also prove detrimental [12,13,14]. Unique degrees of ischemic injury may induce distinct glial reactivity and functional preferences. To fully explore the potential research value of this model, we examined two different levels of PTS models on postnatal day 7 (P7) mice, analyzing the dynamic changes in glial cell and neuronal expression following ischemic injury during brain development. This provides additional options for selecting models to study the development and prevention of neonatal ischemic stroke.

## 2. Materials and Methods

### 2.1. Animals

Wild-type C57BL/6J mice were purchased from the Shanghai SLAC Laboratory, Shanghai, China. The following transgenic mice were used: hGFAP-CreERT2 (The Jackson Laboratory, Bar Harbor, ME, USA. Cat. #: 012849) [15] and Rosa-tdTomato (tdT) (The Jackson Laboratory, Cat. #: 007914) [16]. The presence of a vaginal plug on the first day of pregnancy was recorded as E0.5. Mice had unrestricted access to food and water, and both male and female mice were included in the study. The animals were housed in a controlled animal facility, where they were subjected to a 12 h light/dark cycle and maintained at a room temperature of 23 ± 2 °C. All animals were treated in accordance with the approved protocol established by the Animal Protection and Use Committee of Shanghai Medical College, Fudan University.

### 2.2. Induction of Photothrombotic Stroke

The photothrombotic stroke model was performed similarly to previous studies [9]. Briefly, rose bengal (Sigma-Aldrich, St. Louis, MO, USA) was administered intraperitoneally at a dose of 50 mg/kg body weight to P7 pups, allowing 15 min for dye absorption. The pups were then anesthetized using isoflurane (induction 3.5–4%; maintained at 1.5–2%). Following iodine disinfection, a midsagittal incision was made on the scalp to expose the parietal skull. The target area on the skull (1.5 mm lateral to the bregma) was marked. A black mask with a diameter of 2 mm was placed over the mouse’s head, exposing the target location. The diode-pumped laser (80 mW; 532 nm; YSHINELASER, Zhongshan, China.) was stereotaxically suspended on the target area for 4 min to induce ischemia. The steps for both severe and mild groups are the same, with the difference being that in the severe group, the distance between the laser and the skull is 1 cm, while in the mild group, it is 1.5 cm. After photothrombosis, the scalp was affixed using a biocompatible adhesive, and the pups were carefully placed on a temperature-controlled heating pad set at 37 °C to ensure their optimal recovery before returning to their home cage. Age-matched control mice underwent a midline scalp incision and received an injection of Rose Bengal, but they were not subjected to laser exposure.

### 2.3. Staining 2,3,5-Triphenyltetrazolium Chloride (TTC)

The infarct volume was assessed at 6 h and 1–14 days after PTS. Mice were deeply anesthetized with sodium pentobarbital (50 mg/kg), and their brain tissues were collected and stored in a −20 °C refrigerator for 20 min before being sliced into sections that were 2 mm thick in the coronal plane. The brain sections were then stained with 2% TTC (Sigma-Aldrich) for 30 min at 37 °C in the dark, with the slices being turned evenly every 8 min to ensure even contact with the TTC staining solution. Subsequently, the tissues were fixed in a 4% paraformaldehyde (PFA) solution to ensure optimal tissue hardness. After staining, the infarct appeared white, while the normal brain tissue was stained red. We measured the infarct area using Image J (Version 2.0.0) as described [17,18], and calculated the brain infarct volume percentage (BIVP).

### 2.4. Brain Tissue Processing

After 6 h and 1–14 days following the induction of focal ischemia, mice were perfused with ice-cold phosphate-buffered saline (PBS) and 4% PFA after anesthetization by pentobarbital sodium. The mouse brain tissue was dissected and immediately fixed in 4% PFA at 4 °C overnight. Subsequently, the brain tissue was dehydrated in a solution of 30% sucrose in PBS at 4 °C for three days and then sliced into sections that were 14 μm thick using a Leica cryostat (Wetzlar, Germany). Brain sections were stored in a −20 °C refrigerator until used for immunofluorescence staining.

### 2.5. Immunohistochemistry and Imaging

Immunohistochemistry was performed as previously described [19]. Briefly, brain sections were washed with 0.1% PBS-Triton X-100 and subsequently blocked with 5% bovine serum albumin (BSA) in PBS-Triton X-100 solution. Following overnight incubation at 4 °C with primary antibodies diluted in the blocking solution under gentle agitation, the sections were washed and then incubated for 2 h with corresponding secondary species-specific anti-bodies conjugated with Alexa Fluor 488, 555, or 647 (Jackson ImmunoResearch, Shanghai, China). Nuclei were counterstained using Hoechst 33342 (1 μg/mL, Sigma Aldrich, Shanghai, China). The following primary antibodies were used: GFAP (Cat. #: G3893, mouse, 1:1000, Thermo Scientific, Waltham, MA, USA), PCNA (Cat. #: sc-56, mouse, 1:1000, Santa Cruz, CA, USA), Aldh1L1 (Cat. #: mabn495, mouse, 1:500), Olig2 (Cat. #: AB9610, rabbit, 1:1000, Thermo Scientific), IBA1 (Cat. #: 019-19741, rabbit, 1:1000, Waco), P2Y12 (Cat. #: AS-55043A, rabbit, 1:500, Anaspec, CA, USA), CD68 (Cat. #: OB-PRB044-T, rabbit, 1:300, Oasis).

The immunofluorescence staining slices were imaged using an inverted confocal laser scanning microscope (Confocal Leica SP8 inverted, Shanghai, China) at a resolution of 1024 × 1024 pixels. Stacks of consecutive confocal images were acquired with a 40× objective at intervals of 4.5 μm (Z-step size: 1.5 μm, number of steps: 3) and sequentially captured using two lasers (helium/neon 594 or 647 nm and argon 488 nm). Subsequently, the sequential Z-stack images were converted to maximum intensity projections utilizing ImageJ software version 2.0.0. Full-brain scanning images of fluorescent slices were obtained using the Evos M7000 imaging system (AMF7000, Invitrogen, Shanghai, China). The cell-counting procedure was conducted by a blinded observer, and all labeled cells within and surrounding the ischemic infarction area in the selected brain sections were enumerated. As for Figure 5D, the cell count range was limited to the lesion core. In the relevant cell counts, the presence of cell nuclei was confirmed through Hoechst 33342 staining. Each mouse had four slides per area of interest, and three or more mice were used for all dependent measurements in each experimental condition.

### 2.6. Statistical Analyses

The statistical analyses were conducted using GraphPad Prism 9.4.1 software. The data were presented as the mean value ± SEM, with sample sizes in the figure legends describing biological replicates. For multiple groups, one-way ANOVA was utilized with Dunnett’s multiple-comparisons test, and a two-tailed Student’s *t*-test was employed for comparisons between groups. A significance level of *p* < 0.05 was considered statistically significant, with * *p* < 0.05; ** *p* < 0.01; *** *p* < 0.001; **** *p* < 0.0001, and n.s. indicating not significant.

## 3. Results

### 3.1. Ischemic Infarction Evaluated by TTC Staining

**Figure 1:**
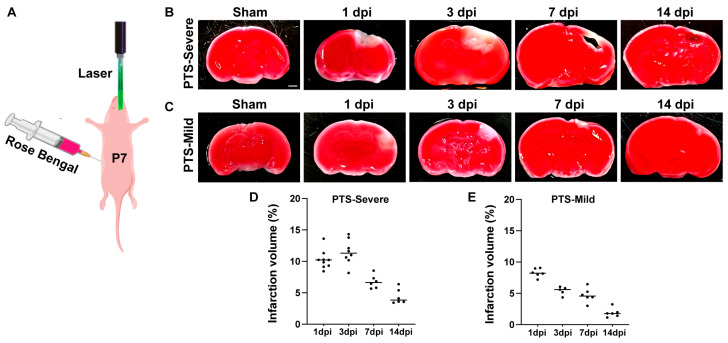
The infarction development in mouse brains after two different degrees of Photothrombotic Stroke (PTS). (**A**) A schematic representation of the PTS experiment at P7 mice. (**B**,**C**) TTC staining of brain tissue in the severe group and mild group. The red area represents normal brain tissue, and the pale area indicates the ischemic infarcted area. Scale bar, 1 mm. (**D**,**E**) The percentage of cerebral infarction volume in mice from the severe group and mild group (*n* ≥ 5 mice).

To examine the progression of ischemic lesions after early postnatal stages, mice with varying degrees of ischemic injury were sacrificed at 1, 3, 7, and 14 days following photothrombotic stroke. The brain sections were then subjected to TTC staining, a commonly used method for demonstrating brain tissue viability (Figure 1A–C).

In the severe group, TTC staining clearly outlined the lesions from the surrounding ischemic core region one day post-infarct (dpi) (Figure 1B). Between 1 and 3 dpi, there was an increase in the lesion area in the cortex. By 7 dpi, TTC images revealed relatively large cavitary lesions in the cortex, with the tissue surrounding the cavity identified as TTC-negative (Figure 1B,D). After 14 days of ischemic injury, the lesion area showed a significant reduction, with a cavity visible in the lesion core. The brain tissue surrounding the cavity exhibited positive staining for TTC, indicating the reaction of the TTC solution with dehydrogenase and the restoration of brain tissue viability (Figure 1B).

In the mild group, similar to the severe PTS group, the lesion was observed one day after ischemic injury (Figure 1C). However, the lesion area in the mild group continued to diminish from 3 to 7 days, with most of the tissues regaining cell vitality at 14 dpi, and only small focal patches of the lesion were visible (Figure 1C,E).

### 3.2. Reactivity and Proliferation of Immature Astrocytes after Ischemic Infarction

**Figure 2:**
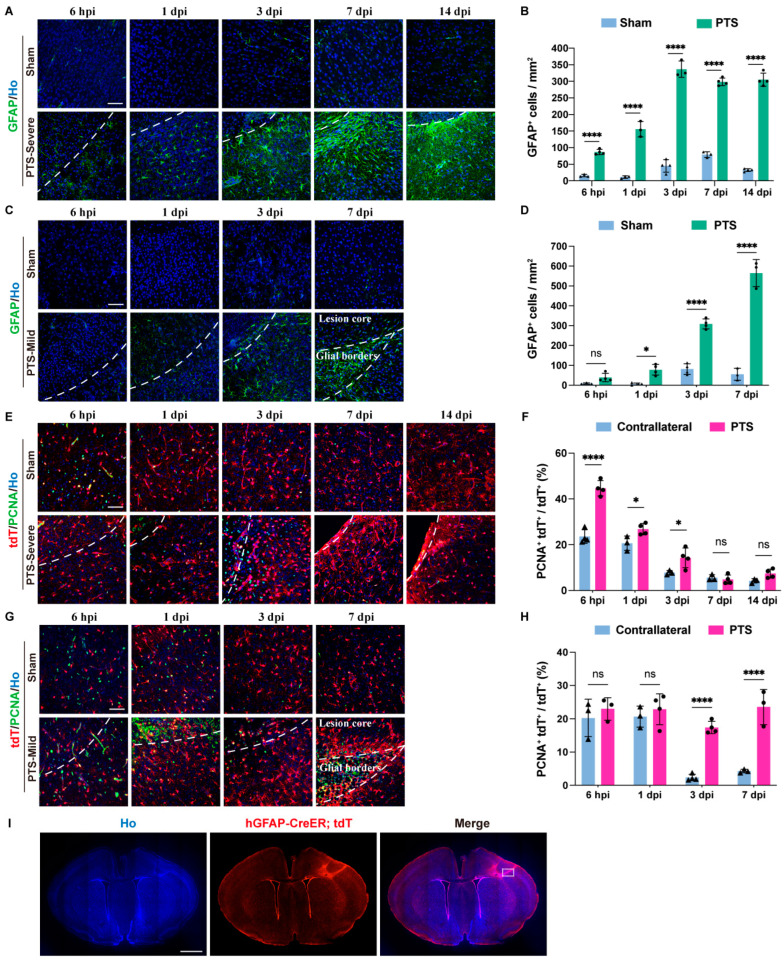
Reactivity and proliferation of astrocytes following ischemic injury at P7 in the mouse cortex. (**A**) Immunostaining of GFAP in the severe group at 6 hpi, 1, 3, 7 and 14 dpi after PTS. The infarction region of the cortex were delineated by white dashed lines. (**B**) Quantification of GFAP+ cells in the severe group at 6 hpi, 1, 3, 7 and 14 dpi after PTS (*n* ≥ 3 mice). (**C**) Immunostaining of GFAP in the mild group at 6 hpi, 1, 3 and 7 dpi after PTS. (**D**) Quantification of GFAP+ cells in the mild group at 6 hpi, 1, 3 and 7 dpi after PTS (*n* ≥ 3 mice). (**E**) Immunostaining of PCNA and tdT in the severe group at 6 hpi, 1, 3, 7 and 14 dpi after PTS. (**F**) Quantification of the ratio of PCNA+tdT+ cells to total tdT+ cells in the severe group (*n* ≥ 3 mice). (**G**) Immunostaining of tdT and PCNA in the mild group at 6 hpi, 1, 3 and 7 dpi after PTS. (**H**) Quantification of the ratio of PCNA+ tdT+ cells to total tdT+ cells in the mild group (*n* ≥ 3 mice). (**I**) Immunostaining of tdT in the mild group at 7 dpi. The analysis area in G is within the white dotted box. Ho, Hoechst 33342 staining. Scale bars, 50 μm in (**A**,**C**,**E**,**G**); 1 mm in I. n.s., not significant; * *p* < 0.05; **** *p* < 0.0001. The symbols represent the sample sizes in the figure legends describing biological replicates.

To understand the dynamic changes in immature astrocytes under two different degrees of ischemic injury, we observed the reactivity of astrocytes through immunofluorescence staining of GFAP. In the severe group, the number of GFAP+ cells indicated an astrocyte reactive response in the ischemic lesion core beginning 6 h post ischemic injury (6 hpi). This response formed an astrocyte border around 3 dpi and persisted until 14 dpi. From 7 dpi to 14 dpi, reactive astrocytes developed soma hypertrophies and protrusions, and cells in proximity to the lesion core polarized and extended particularly long processes (Figure 2A,B). In the mild group, the number of GFAP+ cells significantly increased from 1 dpi to 3 dpi after PTS. GFAP immunofluorescence staining revealed that a dense astrocyte border was formed adjacent to the deep cortical layers at the lesion edge at 7 dpi (the longest time point studied in this group). However, compared with the severe group, the borders had lower astrocyte density (Figure 2C,D).

We used hGFAP-CreERT2; Rosa-tdT transgenic mice to investigate the impact of PTS on the development of immature astrocytes. The hGFAP-CreERT2 mouse line is a powerful tool for gene-targeting of astrocytes in the cortex [20,21]. To determine the proliferation of immature astrocytes, we performed immunostaining with PCNA, a marker of cell proliferation, to evaluate the proliferation of tdT+ cells post-PTS during development. Under sham conditions, 20% of the tdT+ cells at P7–P8 exhibited proliferation. The ratio of tdT+PCNA+ to tdT+ fell below 5% after 3 dpi (Figure 2F,H). In the severe group, the ratio of tdT+PCNA+ to tdT+ showed a significant increase in proliferative tdT+ cells post-PTS at 6 hpi. The ratio decreased at 3 dpi compared to 1 dpi, but it remained significantly higher than the sham group. The proliferation levels of tdT+ cells returned to normal at 7 dpi and 14 dpi (Figure 2E,F). In the mild group, the number of proliferative tdT+ cells significantly increased post-PTS at 3 dpi and remained elevated until 7 dpi (Figure 2G,H). Furthermore, at 7 dpi, we observed that the cortical tissue of the lesion core remained structurally intact, and tdT+ cells redistributed within the lesion core (Figure 2I).

### 3.3. Dynamic Changes in Immature Astrocytes after Ischemic Infarction

**Figure 3:**
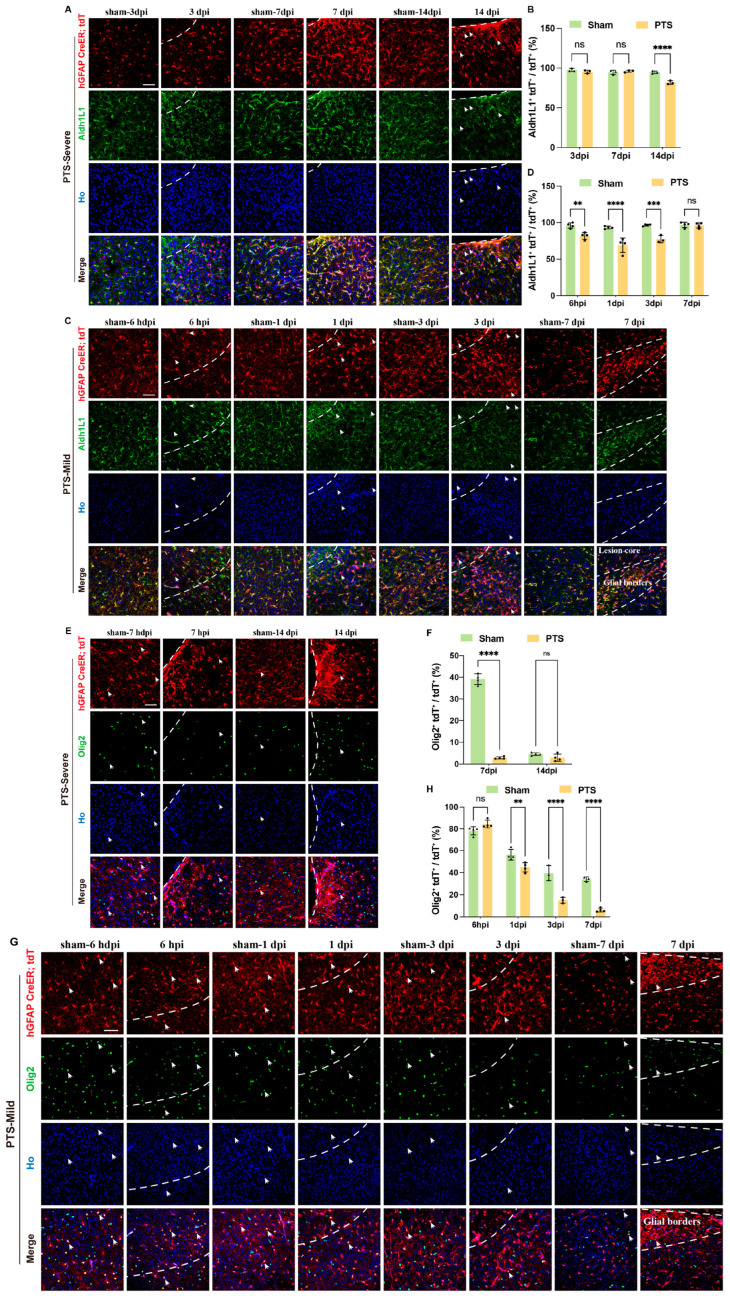
Effects of ischemic infarction on immature astrocytes at P7 in the mouse cortex. (**A**) Immunostaining of tdT and Aldh1L1 in the severe group at 3, 7 and 14 dpi after PTS. The infarction region of the cortex were delineated by white dashed lines. Arrows indicate tdT+ Aldh1L1-cells. (**B**) Quantification of the ratio of tdT+ Aldh1L1+ cells to tdT+ cells in the severe group at 3, 7 and 14 dpi after PTS (*n* = 3 mice). (**C**) Immunostaining of tdT and Aldh1L1 in the mild group at 6 hpi, 1, 3 and 7 dpi after PTS. Arrows indicate tdT+ and Aldh1L1-cells. (**D**) Quantification of the ratio of tdT+ Aldh1L1+ cells to total tdT+ cells in the mild group at 6 hpi, 1, 3 and 7 dpi after PTS (*n* ≥ 3 mice). (**E**) Immunostaining of tdT and Olig2 in the severe group at 7 and 14 dpi after PTS. Arrows indicate cells double-labeled with tdT and Olig2. (**F**) Quantification of the ratio of tdT+Olig2+ cells to total tdT+ cells in the severe group at 7 and 14 dpi after PTS (*n* = 4 mice). (**G**) Immunostaining of tdT and Olig2 in the mild group at 6 hpi, 1, 3 and 7 dpi after PTS. Arrows indicate Olig2 and tdT double-labeled cells. (**H**) Quantification of the ratio of Olig2+tdT+ cells to total tdT+ cells in the mild group at 6 hpi, 1, 3 and 7 dpi after PTS (*n* = 3 mice). Ho, Hoechst 33342 staining. ** *p* < 0.01; *** *p* < 0.001; **** *p* < 0.0001; n.s., not significant. Scale bars, 50 μm in (**A**,**C**,**E**,**G**). The symbols represent the sample sizes in the figure legends describing biological replicates.

Our recent research has shown that when hGFAP-CreERT2; Rosa-tdT mice are intraperitoneally injected with tamoxifen at postnatal day 4 (P4), over 95% of tdT-positive cells co-localize with Aldh1L1 at P7 [19]. To track the dynamic changes of immature astrocytes, we conducted the immunostaining of sections at different time points using antibodies specific for Aldh1L1 and Olig2. In the severe group, the ratio of Aldh1L1+tdT+ to tdT+ cells was significantly lower at 14 dpi, but not at 3 dpi and 7 dpi compared to the sham mice (Figure 3A,B). In the mild group, the ratio of Aldh1L1+tdT+ to tdT+ cells around the lesion significantly decreased from 6 hpi and continued until 3 dpi. At 7 dpi, this ratio returned to normal (Figure 3C,D). During cortical development, Olig2 is transiently expressed in immature developing astrocytes at neonatal stages and is progressively downregulated in astrocytes at late postnatal stages [22,23]. In the severe group, the proportion of tdT+Olig2+ to tdT+ declined substantially at 7 dpi and persisted until 14 dpi (Figure 3E,F). Under sham conditions in the mild group, 80% of the tdT+ cells in hGFAP-CreERT2; Rosa-tdT mice at P7 were co-labeled with Olig2. After that, the proportion of tdT+ Olig2+ to tdT+ slowly declined, and at P21 the ratio fell to a very low level. The ratio of tdT+ Olig2+ to tdT+ showed a slight decrease in developing astrocytes expressing Olig2 after PTS at 1 dpi, followed by a dramatic decrease at 3 dpi and 7 dpi (Figure 3G,H).

### 3.4. Dynamic Activation of Microglia Cells after Early Postnatal PTS

**Figure 4:**
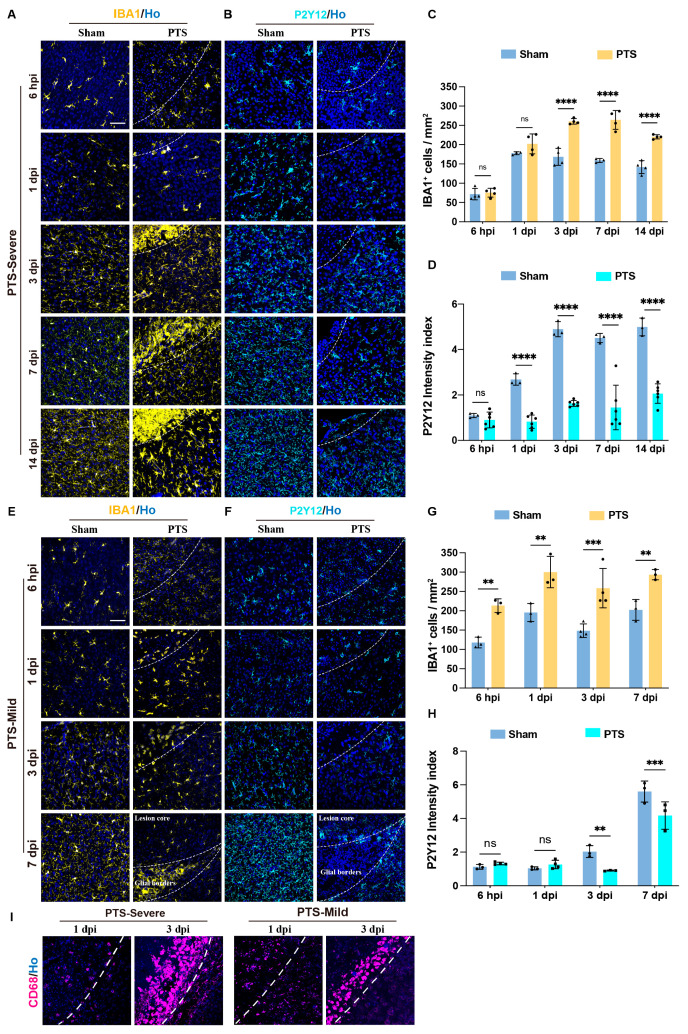
Dynamic changes in the expression of different microglia markers following PTS at P7 in the mouse cortex. (**A**,**B**) Immunostaining of IBA1 or P2Y12 in the severe group at 6 hpi, 1, 3, 7 and 14 dpi after PTS. The infarction region of the cortex were delineated by white dashed lines. (**C**) Quantification of IBA1+ cells in the severe group at 6 hpi, 1, 3, 7 and 14 dpi after PTS (*n* ≥ 3 mice). (**D**) The P2Y12 intensity index in the severe group at 6 hpi, 1, 3, 7 and 14 dpi after PTS (*n* ≥ 3 mice). (**E**,**F**) Immunostaining of IBA1 or P2Y12 in the mild group at 6 hpi, 1, 3 and 7 dpi after PTS. The infarction region of the cortex were delineated by white dashed lines. (**G**) Quantification of IBA1+ cells in the mild group at 6 hpi, 1, 3 and 7 dpi after PTS (*n* ≥ 3 mice). (**H**) The P2Y12 intensity index in the mild group at 6 hpi, 1, 3 and 7 dpi after PTS (*n* ≥ 3 mice). (**I**) Representative images of CD68 immunostaining in severe group and mild group at 1 and 3 dpi after PTS. The infarction region of the cortex were delineated by white dashed lines. Ho, Hoechst 33342 staining. ** *p* < 0.01; *** *p* < 0.001; **** *p* < 0.0001; n.s., not significant. Scale bars, 50 μm in (**A**,**E**,**I**). The symbols represent the sample sizes in the figure legends describing biological replicates.

Microglial cells are the primary immune cells within the brain’s parenchyma [24]. To explore the dynamic responses of microglia following early postnatal PTS, we performed IBA1, P2Y12, and CD68 immunostaining (Figure 4A,E,I). IBA1 is capable of labeling microglia/macrophages, while P2Y12 serves as a valuable marker for identifying microglia under physiological conditions [25,26]. CD68 acts as a general marker for activated phagocytic microglia/macrophages [27]. 

Under sham conditions, there was a sparse presence of IBA1+ cells with fewer cell ramifications in the cortex at P7. Although the number of IBA1 cells increased at P8, the cell ramification remained limited. From P10, IBA1+ cells were widely distributed in the cortex with a stable quantity and dense ramification (Figure 4C,G). Following early postnatal PTS, the severe group exhibited a rapid increase in IBA1 expression within 6 hpi, and at 1 dpi, the IBA1+ cells at the lesion edge displayed an amoeboid phenotype. At 3 dpi, a dense microglia boundary formed at the edge of the lesion, which persisted until 14 dpi (Figure 4A,C). In the mild group, similarly, IBA1+ cells rapidly responds after 6 hpi, with a significant increase in cell numbers and ramifications. The number of IBA1+ cells significantly increased from 6 hpi to 7 dpi. At 1 to 3 dpi, cells exhibited an amoeboid-like phenotype and gathered at the edges of the lesion. The inflammatory response in this group was less intense than that in the severe group. At 7 dpi, a denser microglial boundary could be observed at the edge of the lesion (Figure 4E,G).

In the sham conditions, P2Y12+ cells were sparsely distributed in the cerebral cortex at P7, with the density index gradually increasing from P8 to P10 and reaching a stable level (Figure 4D,H). In the severe group, there was a significant decrease in the expression of P2Y12+ cells from 1 dpi, persisting until 14 dpi. Additionally, at 7 and 14 dpi, there was noticeably less distribution of P2Y12 around the edges of the lesion (Figure 4B,D). In the mild group, there was a significant decrease in P2Y12 density at 1 and 3 dpi. However, at 7 dpi, P2Y12 was only distributed in areas outside of the glial scar region, including tissues that were once located at the lesion core and surrounding tissues (Figure 4F,H).

CD68 (cluster of differentiation 68, CD68), as a lysosomal marker, is recognized as a general marker for activated phagocytic microglia/macrophages. To assess the distribution of the microglial boundary, we examined CD68 expression around lesions in both severe and mild groups at 1 dpi and 3 dpi. Our results indicated a lower number of CD68 cells expressed at 1 dpi. By 3 dpi, a distinct CD68+ microglial cell boundary was evident around the lesion edge, with thinner cells observed in the mild group compared to the severe group (Figure 4I).

### 3.5. Neuronal Regeneration and Tissue Repair around the Ischemic Area

**Figure 5:**
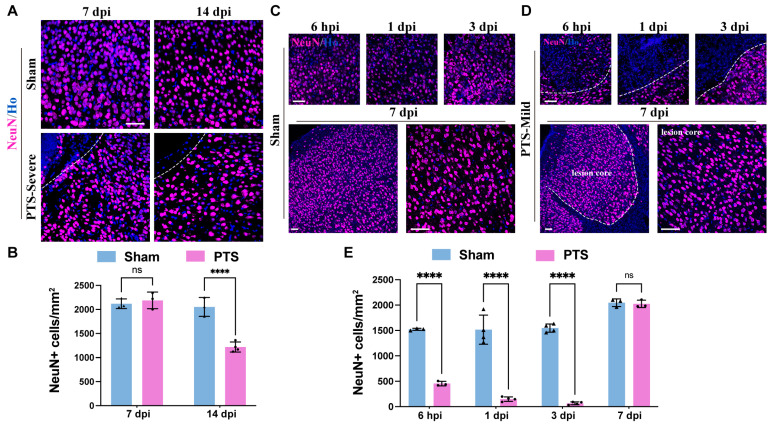
Regeneration of neurons surrounding the ischemic infarction and tissue repair. (**A**) Immunostaining of NeuN in the severe group at 7 and 14 dpi after PTS. The infarction region of the cortex is delineated by white dashed lines. (**B**) Quantification of NeuN+ cells in the severe group at 7 and 14 dpi after PTS (*n* ≥ 3 mice). (**C**,**D**) Immunostaining of NeuN in the mild group at 6 hpi, 1, 3 and 7 dpi after PTS. The infarction region of the cortex is delineated by white dashed lines. (**E**) Quantification of NeuN+ cells in the mild group at 6 hpi, 1, 3 and 7 dpi after PTS (*n* = 4 mice). Ho, Hoechst 33342 staining. n.s., not significant; **** *p* < 0.0001. Scale bars, 50 μm in (**A**,**C**,**D**). The symbols represent the sample sizes in the figure legends describing biological replicates.

To evaluate the neurogenesis following PTS, we conducted immunostaining to examine the expression of NeuN around the ischemic area. In the severe group, brain sections were analyzed at 7 dpi and 14 dpi, revealing a scarcity of NeuN immunoreactive neurons within the lesion core at 7 dpi. Additionally, there was a significant decrease in the number of neurons in the surrounding area at 14 dpi, indicating neuronal loss in the adjacent tissues (Figure 5A,B). Conversely, in the mild group, degenerating neurons stained by NeuN were observed as early as 6 hpi. At 1 and 3 dpi, there were almost no NeuN+ neurons within the core of the lesion. However, at 7 dpi, NeuN+ cells redistributed outside the glial boundary, encompassing both the core of the lesion and surrounding healthy tissue, with no significant difference in the number of NeuN+ cells between the lesion core and surrounding tissues (Figure 5C–E).

## 4. Discussion

Perinatal ischemic stroke, also referred to as neonatal ischemic stroke, can result in hemiplegia or other symptoms of neurological disorders, representing a significant cause of death and disability in premature infants [1]. While numerous factors contribute to the manifestation of these symptoms, vascular obstruction emerges as the primary causative factor [28]. The PTS model is utilized to simulate the occlusion of small cerebral vessels and is particularly suitable for studying the cellular and molecular mechanisms underlying neuroprotection and neuro-regeneration [10]. While the perinatal PTS model is mainly applied in rats, successful attempts have been made to model PTS in P7 mice [9]. However, the molecular response and tissue repair following PTS in P7 mice remain unclear. In this study, by using two different degrees of an ischemic injury mouse model, we showed the dynamic changes in glial response and neuronal regeneration during the acute phase of the PTS model in neonatal mice. This provides a valuable model reference for investigating the cellular and molecular mechanisms of neuroprotection and neural regeneration associated with neonatal ischemic stroke.

In the severe group, a dense border of astrocytes and microglia was observed within three days post-infarction, leading to the formation of a permanent cortical cavity and neuronal loss in the surrounding tissues. In contrast, the mild group showed a relatively sparse arrangement of glial borders, intact cortical tissue, and restoration of viability in the brain tissue beyond the glial boundary. These results revealed distinct differences of the glial response and neuronal regeneration between the severe and mild PTS models. The presence of two distinct degrees of ischemic injury offers an opportunity to explore the causal relationship between the spatiotemporal characteristics of glial cells and disease progression/recovery. Thus, it can aid in the development of research strategies for treating neonatal ischemic injury, with an emphasis on reducing detrimental effects and encouraging a beneficial glial response.

The development of astrocytes, including their production, migration, and maturation, primarily occurs during the first three to four weeks after birth [29]. The mechanism and potential for astrocyte repair after injury differ greatly between neonatal and adult astrocytes [30]. Our results showed changes in the molecular expression levels of astrocytes during development, providing new evidence for the different molecular responses of astrocytes during developmental and mature stages in response to ischemic injury. Aldh1L1 has been found to be highly expressed in CNS parenchymal astrocytes [31]. Our previous research has also demonstrated that within the cortex of hGFAP-CreERT2; Rosa-tdT transgenic mice, a remarkable co-localization of over 95% tdT+ cells with Aldh1L1 was observed. [19]. Our findings indicate that after PTS at the cortex of P7 mice, the expression level of Aldh1L1 significantly decreases in developing astrocytes. Notably, this change appeared earlier in the mild group than in the severe group, suggesting its potential relation to tissue repair in perinatal mice after ischemic injury.

During cortical development, Olig2 exhibits transient expression in immature astrocytes at neonatal stages and undergoes progressive downregulation in late postnatal astrocytes [22,23]. Astrocytes in the Olig2-ablated cortex exhibit sustained upregulation of GFAP in the superficial layers [32]. Our study found that ischemic injury at postnatal stages induced the upregulation of GFAP expression and concomitant downregulation of Olig2 expression in astrocytes. However, the causal relationship between these two events remains unclear.

Microglia play a crucial role in regulating the number of neuronal precursors by engulfing neural precursor cells during cortical development [33,34]. The majority of microglia in the postnatal cerebral cortex exhibit an activated morphology and express markers indicative of activation. During periods of neurogenesis, there are relatively few IBA1+ cells present in the cortical plate, and their distribution is consistent with that observed in the developing neocortex of humans, macaques, and rats [33,35]. Our research indicates that IBA1+ cells in mice follow a similar expression pattern, with a limited number of IBA1+ cells present in the early postnatal mouse cortex, predominantly displaying a polarized morphology.

Despite the existence of various models for neonatal ischemic stroke, the lack of animal models for perinatal ischemic stroke that accurately mirror the mechanisms underlying both acute and long-term deficits in onset and progression remains a challenge. In this study, we observed varying outcomes in the mild and severe groups. Examining the mechanisms driving glial cell reactivity and functionality following ischemic injury in two pathological states, as well as contrasting the processes of neuronal regeneration and regeneration failure between these two conditions, may provide new insights into disease progression control and tissue repair promotion following ischemic damage.

In conclusion, our study has demonstrated a series of dynamic changes in glial cells and neuronal expression following varying degrees of ischemic injury in a mouse PTS model. Furthermore, our findings have provided insights into the potential influence or alteration of neuroglia cell development during the pathogenesis of ischemic brain damage. These discoveries offer new perspectives on the cellular and molecular mechanisms underlying neuroprotection and neural regeneration following ischemic brain damage.

## Data Availability

The data presented in this study are openly available.
